# Supplementary material for: Fast Estimation of L1-Regularized Linear Models in the Mass-Univariate Setting
Source: Neuroinformatics. 2020 Sep 15;19(3):385–92. doi: 10.1007/s12021-020-09489-1 (PMC8233242; doi:10.1007/s12021-020-09489-1)
Supplement: Supplementary file 1 — (PDF 694 kb) [file 12021_2020_9489_MOESM1_ESM.pdf]

Supplementary Material

Estimation of memory usage

Memory usage can be estimated using the following heuristics:

lasso_mex (CPU): Number of floating point values:

$$\#FP \approx np + nv + p^2 + pv + 1.5nv(N_\lambda + 2)b\_f$$

with b_f being the `buffer_factor`, and N_λ being the length of the lambda sequence.
In `lasso_mex`, floating point format is 64-bit double precision, thus memory usage in GB is

$$8 \cdot \#FP/10^9$$

For example, for benchmark A this translates into a memory usage of approximately 7.95 GB.

lasso_mexcuda (GPU): Number of floating point values:

$$\#FP \approx 2np + 7p \cdot b\_s$$

with b_s being the `buffer_size`. In `lasso_mexcuda`, floating point format is 32-bit single precision, thus GPU memory usage in GB is

$$4 \cdot \#FP/10^9$$

For example, for benchmark A this translates into a GPU memory usage of approximately 2.31 GB.

lasso_gpu (GPU): Number of floating point values:

$$\#FP \approx 2np + 8p \cdot b\_s$$

with b_s being the `buffer_size`. In `lasso_gpu`, floating point format is 32-bit single precision, thus GPU memory usage in GB is

$$4 \cdot \#FP/10^9$$

For example, for benchmark A this translates into a GPU memory usage of approximately 2.63 GB.

Computation time as a function of CPU/GPU cores

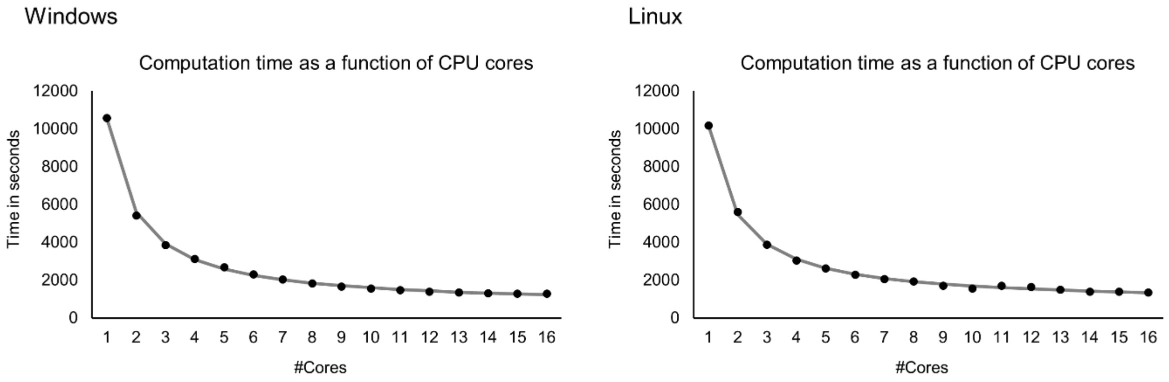

Supplementary Figure 1. Computation time as a function of CPU cores, using the function `lasso_mex` on benchmark A. Black points correspond to actual data points of the benchmark, and the gray lines correspond to Amdahl's law (Amdahl 1967, see below) with $p = 0.944$ (Windows) and $p = 0.927$ (Linux), indicating that the degree of parallelization achieved by `lasso_mex` was 94.4% and 92.7%, respectively.

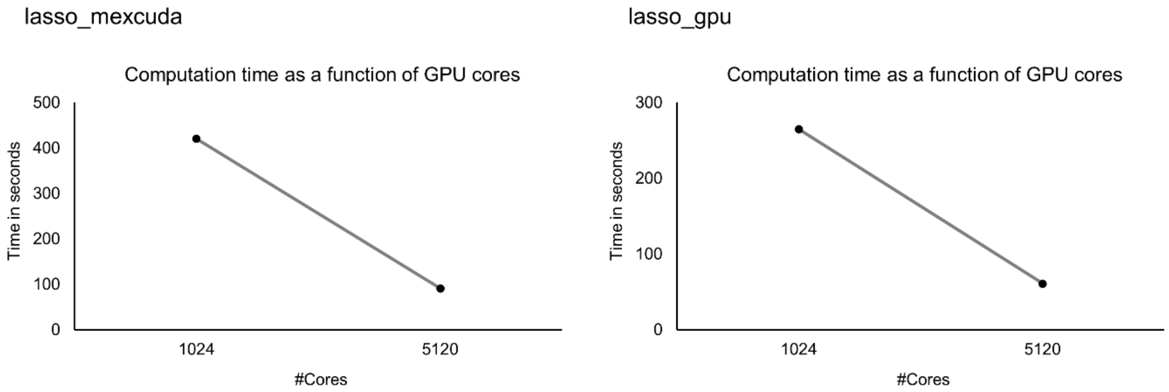

Supplementary Figure 2. Computation time as a function of GPU cores, using the functions `lasso_mexcuda` and `lasso_gpu` on benchmark A. Black points correspond to actual data points of the benchmark, and the gray lines correspond to Amdahl's law (Amdahl 1967, see below) with p=0.99998 (`lasso_mexcuda`) and p=0.99996 (`lasso_gpu`), indicating that the degree of parallelization was 99.998% and 99.996%, respectively. Note that these values are based on a comparison of two different GPU devices and thus can only serve as approximate estimations.

Estimation of the degree of parallelization was based on Amdahl's law (Amdahl 1967), with S being the speed-up factor, c being the number of processor cores, and p the degree of parallelization:

$$S(c, p) = \left(1 - p + \frac{p}{c}\right)^{-1}$$

Amdahl, G.M. (1967). Validity of the single processor approach to achieving large scale computing capabilities. In Proceedings of the April 18-20, 1967, Spring Joint Computer Conference on - AFIPS '67 (Spring)

Supplementary Tables

300 x 5000	Windows	Linux
OLS	-	-
Ridge	-	-
gpuArray	277.8	265.0
cuBLAS	431.6	420.5
OpenMP	1290.8	1351.9
C++	10567.6	10175.3
Matlab	32777.4	17952.3

300 x 200	Windows	Linux
OLS	0.18	0.15
Ridge	0.17	0.16
gpuArray	3.51	2.26
cuBLAS	1.29	0.98
OpenMP	0.85	0.69
C++	4.91	5.10
Matlab	395.42	217.65

Supplementary Table 1. Computation times for benchmarks A and B in seconds.

300 x 5000	Tesla V100	Quadro P2000
gpuArray	60.9	265.0
cuBLAS	91.5	420.5
Matlab	17952.3	17952.3

Supplementary Table 2. Computation times in seconds for benchmarks A on two different GPU devices.
